# Impact of Coronavirus Disease 2019 on the Diagnosis and Treatment of Pancreatic Cancer: An Observational Cohort Study

**DOI:** 10.5152/tjg.2024.23563

**Published:** 2024-05-01

**Authors:** Hyung Ku Chon, Eun Jee Lee, Yun Chae Lee, Seong-Hun Kim

**Affiliations:** 1 Division of Biliopancreas, Department of Internal Medicine, Wonkwang University Medical School and Hospital, Iksan, Korea; 2 Institute of Wonkwang Medical Science, Iksan, Korea; 3 Research Institute of Nursing Science, Jeonbuk National University College of Nursing, Jeonju, Korea; 4 Division of Gastroenterology, Department of Internal Medicine, Jeonbuk National University Medical School, Jeonju, Korea; 5 Research Institute of Clinical Medicine of Jeonbuk National University-Biomedical Research Institute of Jeonbuk National University Hospital, Jeonju, Korea

**Keywords:** COVID-19, pancreatic cancer, adult, change management, neoplasm staging

## Abstract

**Background/Aims:**

Early pancreatic cancer diagnosis is crucial for timely intervention and better outcomes. The coronavirus disease 2019 (COVID-19) pandemic has disrupted routine health care globally. The COVID-19 pandemic has disrupted routine health care globally. This study aimed to evaluate the impact of COVID-19 on the diagnosis and treatment of pancreatic cancer.

**Materials and Methods:**

This retrospective study compared pancreatic cancer patients from 2 tertiary hospitals in pre and COVID-19 periods. Pre-COVID-19 period spanned from January 1, 2018, to January 19, 2020, while the COVID-19 period extended from January 20, 2020, to December 31, 2021.

**Results:**

A total of 542 patients were included. In the pre-COVID-19 period, 280 new cases of pancreatic cancer were enrolled, compared to 262 during COVID-19. The annual incidence rates were 136.63 and 134.50 patients, respectively. The median age was significantly lower during COVID-19 (71.5 years) compared to pre-COVID-19 (77 years) (*P* < .001). In subgroup analyses for stage 3 and 4, the proportion of stage 4 pancreatic cancer increased during COVID-19 (*χ*^2^ = 5.53, *P* = .019), and the COVID-19 group had younger diagnoses, better performance status, more surgery, higher use of FOLFIRINOX chemotherapy, fewer hospital referrals, and better median overall survival compared to the pre-COVID-19 group.

**Conclusion:**

This study revealed changes in the characteristics and treatment of patients diagnosed with pancreatic cancer during the COVID-19 pandemic. Although further large-scale research is necessary, the findings of this study can function as foundational data for formulating policies for the management of patients with pancreatic cancer during future pandemics of other infectious diseases.

**Main Points**

- In the current study, no significant differences were observed in newly diagnosed with pancreatic cancer patients between the pre-coronavirus disease 2019 (COVID-19) and COVID-19 periods in terms of incidence rate, tumor stage, or median overall survival. However, the COVID-19 group tended younger age, have a better Eastern Cooperative Oncology Group performance status, compared to the pre-COVID-19 group.
- The proportion of stage 4 pancreatic cancer patients was higher during the COVID-19 period compared to pre-COVID-19 in subgroup analyses for stage 3 and 4.
- During the COVID-19 period, patients with stage 3 and 4 had more chemotherapy with FOLFIRINOX and better median overall survival compared to those in the pre-COVID-19 period.

## Introduction

The coronavirus disease 2019 (COVID-19) outbreak has significantly impacted health-care systems worldwide.^1-3^ To better allocate resources and reduce the viral transmission risk, many health-care facilities were obliged to postpone or cancel non-urgent procedures, surgeries, and appointments.^4,5^ Several guidelines recommended individuals to avoid hospital visits and surgeries during the COVID-19 pandemic, unless absolutely necessary.^6,7^

Pancreatic cancer is a highly aggressive and lethal malignancy, with a 5-year survival rate of only 13.9%.^8^ Treatment with radical surgical resection and adjuvant or neoadjuvant chemotherapy is important to improve survival outcomes. Therefore, the early diagnosis of pancreatic cancer is crucial, as the disease progresses rapidly. However, the fear of contracting COVID-19, the burden of the pandemic on health-care systems, and the implementation of social distancing measures may have resulted in a decreased number of people seeking medical care, including those experiencing symptoms or seeking cancer evaluations. The focus of health-care resource allocation shifted towards managing COVID-19 cases, diverting attention and resources from routine cancer diagnosis and care. This may have impacted the diagnosis and treatment of pancreatic cancer, including less and delayed diagnoses, change in therapeutic approaches and overall survival rates. Evaluating the impact of the pandemic on the diagnosis and treatment of this malignancy can provide valuable insights into the potentially occurred changes in patient outcomes and health-care delivery; however, research examining the effects of the COVID-19 pandemic on these factors is lacking.

In this study, we retrospectively compared the characteristics, treatment trends, incidence rate, tumor staging, as well as the initial treatment approach among newly diagnosed pancreatic cancer patients before and after the onset of COVID-19. To our knowledge, this is the first study to examine such data in Korea.

## Materials and Methods

In this study, medical records of consecutive patients with newly diagnosed pancreatic cancer from January 2018 to December 2021 were retrospectively collected from 2 academic tertiary-care hospitals, allowing the comparison of patients before and after the COVID-19 outbreak. The pre-COVID-19 period was defined as the one between January 1, 2018, and January 19, 2020, while the post-COVID-19 period was that between January 20, 2020, and December 31, 2021. This division was based on the date of the first confirmed case of COVID-19 in South Korea, which occurred on January 20, 2020. The patients enrolled in this study were followed up until February 28, 2023. This extended follow-up period allowed for the collection of long-term data on the outcomes and progression of pancreatic cancer. The inclusion criteria for the study were as follows: (1) patients admitted to the participating tertiary hospitals for evaluation of suspected pancreatic cancer; (2) patients diagnosed with pancreatic adenocarcinoma by cytology or biopsy; (3) patients with characteristic pancreatic cancer findings on 2 or more imaging examinations such as ultrasonography, abdominal computed tomography (CT), abdominal magnetic resonance imaging, positron emission tomography-CT, endoscopic ultrasound, or endoscopic retrograde cholangiopancreatography, in cases in which diagnosis confirmation by cytology or biopsy was difficult; and (4) patients aged 18 years or older. The exclusion criteria were as follows: (1) patients who visited the hospitals after undergoing surgery or chemotherapy for pancreatic cancer at other facilities; (2) patients who were transferred to other centers before diagnostic confirmation at the participating hospitals or without definition of treatment; (3) patients diagnosed with endocrine tumors or carcinomas based on cytology or biopsy. Collected baseline characteristics included age, sex, symptoms present at hospital visits, Eastern Cooperative Oncology Group (ECOG) performance status, and laboratory findings [such as serum levels of hemoglobin (g/dL), albumin (g/dL), carbohydrate antigen 19-9 (CA 19-9) (U/mL), total bilirubin (mg/dL), glycated hemoglobin (HbA1C) (%), and C-reactive protein (CRP) (mg/L)]. Additionally, the CA 19-9 data for patients who underwent biliary drainage were recorded approximately 1 month after the procedure. Pancreatic cancer-related characteristics were determined by tumor size, location, and staging based on the eighth edition of the American Joint Committee on Cancer staging manual. In addition, treatment modalities including surgery, chemotherapy regimens, conservative treatment, transfer to other centers for a second opinion, and survival rates were also obtained. This study was approved by the Research Ethics Review Committee of Jeonbuk National University and Wonkwang University Hopital, respectively (IRB Nos. CUH 2022-05-029-001 and WKUH 2022-05-002). Waiver of informed consent was granted by Human Research Ethics Committee, Faculty of Jeonbuk National University Hospital and Wonkwang University Hospital, because the data have been anonymized.

### Statistical Analysis

Frequency, percentage, median, and range were calculated for subject and pancreatic cancer-related characteristics, and comparisons for the periods before and after the COVID-19 outbreak were performed using the chi-square test (Fisher’s exact test) for categorical variables and the Mann–Whitney *U*-test for continuous variables. Statistical significance for all tests was set at a *P*-value of less than .05. Survival curves were constructed using the Kaplan–Meier method and analyzed using the log-rank test. Univariate and multivariate analyses were performed using the Cox proportional hazards model to analyze the prognostic factors.

## Results

### Characteristics of the Patients

A total of 542 patients were included in the study. In the pre-COVID-19 period, 280 new cases of patients with pancreatic cancer were diagnosed, whereas 262 new cases were diagnosed during the COVID-19 period, resulting in annual incidence rates of 136.63 and 134.50 patients, respectively. The baseline characteristics of the patients according to the periods are presented in Table 1. No significant differences were observed between the 2 groups regarding sex, levels of CA 19-9, total bilirubin or HbA1C, cancer stage, CRP, and tumor location. However, a significant difference in the median age of patients between the pre-COVID-19 (77 years) and COVID-19 groups (71.5 years) (*P *< .001) was found. Additionally, the median hemoglobin and albumin levels tended to be higher in the COVID-19 group than in the pre-COVID-19 group (*P *= .028 and *P =* .009, respectively). Moreover, abdominal pain was more frequently reported as a symptom at the time of hospital visit, and worse ECOG performance statuses were observed in the pre-COVID-19 group than in the COVID-19 group (*P* = .014 and *P* = .011, respectively).

### Treatment Modalities and Survival

**Figure 1.**
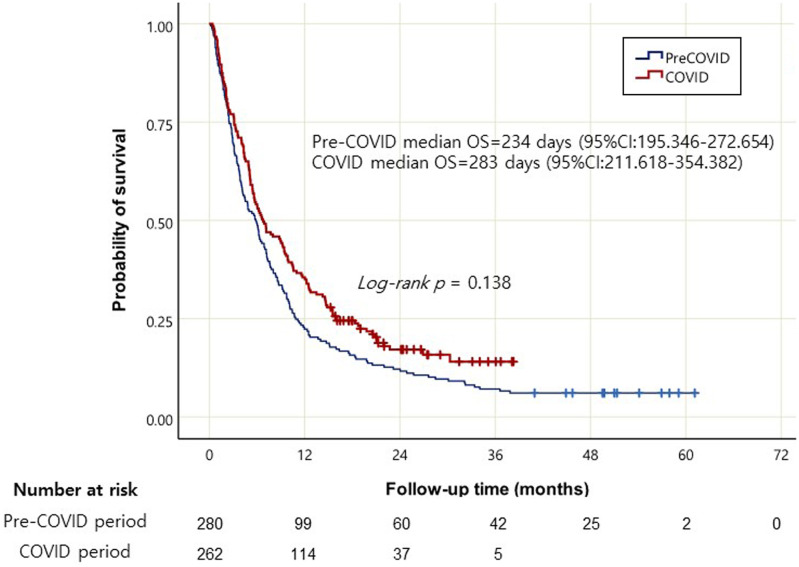
Kaplan–Meier curves of overall survival in patients newly diagnosed with pancreatic cancer in the pre-COVID-19 and COVID-19 periods. COVID-19, coronavirus disease 2019.

Compared to pre-COVID-19 group, the COVID-19 group underwent a higher number of surgeries and received chemotherapy more frequently, while receiving less supportive care and had fewer referrals to other hospitals for a second opinion (Table 2). In the pre-COVID-19 group, gemcitabine plus nab-paclitaxel (Gem + Nab) was chosen more frequently as the initial chemotherapy regimen, whereas there was a significant preference for the FOLFIRINOX regimen (fluorouracil, leucovorin, irinotecan, and oxaliplatin) in the COVID-19 group. The 1, 2, and 3-year survival rates during the pre-COVID-19 period were 35.4%, 21.4%, and 15.0%, respectively. In the COVID-19 group, the rates were 43.5%, 24.5%, and 18.8%, respectively. However, no significant difference was observed in the median overall survival between the 2 groups (Figure 1).

### Comparison Between Patients Diagnosed with Stage 3 and 4 Pancreatic Cancer During the Pre-Coronavirus Disease 2019 and Coronavirus Disease 2019 Periods

In the subgroup analyses of stage 3 and 4 pancreatic cancer cases, Table 3 presents a comparison of the number of patients with stage 3 and 4 pancreatic cancer in the pre- and post-COVID-19 periods. The proportions of patients diagnosed with stage 3 pancreatic cancer were 24.4% in the pre-COVID-19 period and 14.8% in the post-COVID-19 period, whereas for those diagnosed with stage 4, the proportions were 75.6% in the pre-COVID-19 period and 85.2% in the post-COVID-19 period. These findings indicated a higher proportion of patients diagnosed with stage 4 disease in the post-COVID-19 period (*χ*^2^ = 5.53, *P* = .019).

**Figure 2.**
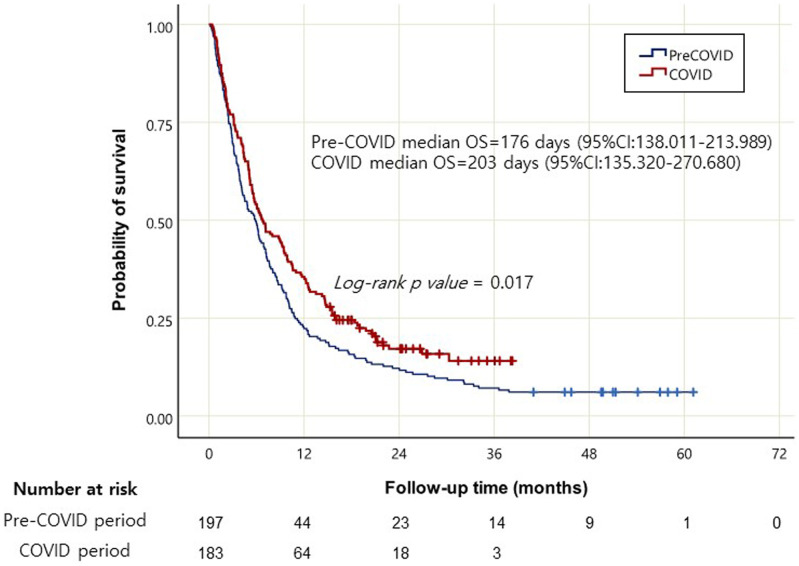
Kaplan–Meier curves of overall survival in patients newly diagnosed with stage 3 and 4 pancreatic cancer in the pre-COVID-19 and COVID-19 periods.COVID-19, coronavirus disease 2019.

The COVID-19 group exhibited a younger age at diagnosis, a better ECOG performance status, a higher percentage of patients receiving chemotherapy with the FOLFIRINOX regimen, and fewer referrals to other hospitals for a second opinion than the pre-COVID-19 group (Table 4). The Kaplan–Meier curve (Figure 2) showed a statistically significant increase in median overall survival among the COVID-19 group with stage 3 and 4 pancreatic cancer (*P* = .017). The median overall survival of the pre-COVID-19 and COVID-19 groups in patients with stage 3 and stage 4 pancreatic cancer was 176 days (95% CI: 138.011-213.989) and 203 days (95% CI: 135.320-270.680), respectively.

### Prognostic Factors for Survival of Patients with Pancreatic Cancer

In the univariate analyses, age, ECOG performance status, tumor staging, elevated CA 19-9 levels, hemoglobin levels, albumin levels, CRP levels, selected treatment modalities, and the presence of fatigue were significantly associated with the survival of pancreatic cancer (Table 5). The multivariate analysis revealed that ECOG performance status, tumor staging, hemoglobin levels, and selected treatment modalities were independent prognostic factors for the survival of pancreatic cancer. However, the presence of the COVID-19 pandemic at the time of diagnosis was not related to survival.

## Discussion

Coronavirus Disease 2019 profoundly impacted the health-care environment, economy, and society.^9,10^ In this large retrospective cohort study, we demonstrated that the proportion of patients diagnosed with stage 4 pancreatic cancer in a population where surgery is challenging was higher in the COVID-19 period than in the pre-COVID-19 period. This finding suggests that delays in the detection of pancreatic cancer during the COVID-19 pandemic may have existed, leading to a higher proportion of patients presenting with more advanced disease stages. We assumed that such phenomena occurred because of various factors, including disruptions in health-care services, reduced access to screening and diagnostic tests, and delays in patients seeking medical attention due to concerns regarding the pandemic. Several studies have shown that the rate of national health checkup examinations has declined significantly in the period.^11-14^

Another notable finding was that in the COVID-19 group, patients were diagnosed at a younger age and presented with better ECOG performance and nutritional statuses, as evidenced by serum hemoglobin and albumin levels. Similar outcomes were observed in the subgroup analyses of patients with stage 3 and 4 disease. These results are presumed to contribute to a higher incidence of chemotherapy and surgery in the COVID-19 group compared to the pre-COVID-19 group. Elderly patients and patients with poor ECOG performance status typically present compromised overall health and reduced immune function, further increasing their susceptibility to COVID-19 infection and its related complications. Therefore, such patients may have been advised or chosen to isolate themselves in nursing homes or at home to reduce their risk of viral exposure. Consequently, they may have been less likely to visit tertiary hospitals for medical evaluation and screening, including those for the diagnosis of pancreatic cancer. Peacock et al^15^ demonstrated that the lower cancer diagnosis rate among older individuals was attributed to people over 80 years of age succumbing to COVID-19 before receiving a cancer diagnosis. Other studies have also demonstrated a decrease in cancer diagnoses among the elderly population during the COVID-19 pandemic.^16,17^

In the current study, there was no significant difference between the 2 groups in terms of overall survival and tumor staging, which is consistent with previous studies.^18,19^ However, in the subgroup analysis, the COVID-19 group with stage 3 and 4 pancreatic cancer exhibited improved median overall survival compared to those diagnosed in the pre-COVID-19 period. This finding is presumed to be due to better performance status and a higher percentage receiving a specific chemotherapy regimen (FOLFIRINOX). Chemotherapy plays a crucial role in improving survival outcomes and managing stages 3 and 4 pancreatic cancer, as it aids in diminishing tumor size, controlling disease progression, and improving quality of life. Various chemotherapy regimens, such as FOLFIRINOX and Gem + Nab, are effective for extending survival and improving overall response rates in patients with advanced pancreatic cancer.^20-22^ Chemotherapy selection is based on multiple factors, including the overall health of the patient, tumor characteristics, and individual treatment goals, as well as emerging clinical evidence and evolving treatment guidelines. The Gem + Nab may be preferred over the FOLFIRINOX regimen for patients with a poorer performance status, older age, and less advanced or aggressive disease, such as tumors with less metastases or a lower tumor burden.^23^ In the current study, the Gem + Nab was the most commonly selected regimen in the pre-COVID-19 group, while there was a significant increase in the employment of the FOLFIRINOX regimen in the COVID-19 group due to differences in age and performance status.

The findings of our study align with several prior research efforts investigating prognostic factors in pancreatic cancer. Consistent with our results, previous studies have highlighted the significance of ECOG performance status and tumor staging in predicting survival outcomes.^24,25^ Moreover, our study reaffirms the importance of hemoglobin and albumin levels as prognostic indicators, in agreement with studies emphasizing the role of nutritional status in influencing pancreatic cancer survival.^26,27^ The observed association between selected treatment modalities and survival further corroborates the impact of treatment strategies on patient outcomes. This is in concordance with literature suggesting that treatment aggressiveness, including surgical interventions and adjuvant therapies, plays a pivotal role in pancreatic cancer prognosis.^28-30^ Interestingly, our study did not find a significant correlation between the presence of the COVID-19 pandemic at the time of diagnosis and pancreatic cancer survival. It is possible that the unique characteristics of pancreatic cancer, along with the pandemic’s dynamic and evolving nature, contributed to this finding. Therefore, our study reinforces well-established prognostic factors in pancreatic cancer while offering a novel perspective by not identifying a direct link between the COVID-19 pandemic and survival outcomes.

In the current study, the number of patients diagnosed with pancreatic cancer was similar within the periods, with 136.63 in the pre-COVID-19 period and 134.50 in the post-COVID-19 period. This differs from a previous study that reported a significant reduction in the number of diagnoses of all carcinomas.^15^ In addition, the COVID-19 group presented fewer referrals to other hospitals for a second opinion compared to the pre-COVID-19 group. These findings can be attributed to the successful implementation of COVID-19 prevention measures in the area of the study institutions. During the study period, the occurrence of COVID-19 cases was well controlled in the regions of the participating hospitals, with fewer than 100 cases per day.^31^ Therefore, the fear of COVID-19 infections is presumed to have led to a decrease in the choice of transferring patients to medical institutions in other areas.

The constant risk of future pandemics similar to COVID-19 necessitates a comprehensive and adaptable approach to managing cancer patients. Cancer has been identified as one of the significant risk factors for mortality in the COVID-19 pandemic.^32^ While specific policies may vary based on the nature of the infectious disease and the state of medical knowledge, telemedicine and remote monitoring can play a crucial role in tracking cancer patients’ vital signs and treatment responses during pandemics. Prioritizing effective vaccination for cancer patients is vital to reduce infection risks. Clear guidelines for risk stratification based on cancer type, stage, and patient health are essential. Educational materials for cancer patients about the risks and preventive measures related to infectious diseases should be created. Encouraging collaboration among health-care institutions, researchers, and international organizations is needed to share information and enhance decision-making and patient outcomes. The policies should be dynamic, aligning with pandemic evolution, based on the latest evidence. Regular reviews ensure ongoing effectiveness.

The COVID-19 pandemic has also impacted the management of chronic gastrointestinal (GI) diseases, leading to treatment delays and interruptions due to resource reallocation, lockdowns, and concerns about in-person health-care visits. Lockdowns and lifestyle changes influenced patients’ dietary habits, potentially affecting conditions like inflammatory bowel disease or irritable bowel syndrome. The stress and anxiety associated with the pandemic also had implications for GI condition management, requiring health-care providers to address psychological aspects as part of the overall strategy.

Our study had several limitations. First, it was based on limited hospital data from a specific region, which may have resulted in differences compared with other regions or health-care systems. Second, the study was observational in nature; therefore, determining a definitive cause-and-effect relationship between the variables is challenging. Third, this study was based on data from a specific time period, which means that the results may vary in the future or during different periods.

In conclusion, this study revealed changes in the characteristics and treatment of patients diagnosed with pancreatic cancer during the pre-COVID-19 and COVID-19 periods. The younger age at diagnosis and improvement in ECOG performance status in patients with pancreatic cancer during the COVID-19 period can be considered evidence that access to medical care for elderly and medically compromised patients became more challenging. The increased number of stage 4 pancreatic cancer cases in a population where surgery is challenging during the COVID-19 period highlights the importance of establishing a robust health-care system that ensures health evaluations, even during a pandemic. Although further large-scale research is necessary, the findings of this study can function as foundational data for formulating policies for the management of patients with pancreatic cancer during future pandemics of other infectious diseases.

## Figures and Tables

**Table 1.** Comparison of the Characteristics of Pancreatic Cancer Patients Between the Pre-Coronavirus Disease (COVID-19) and COVID-19 Periods

Variables	Pre-COVID-19 Period (n = 280)		COVID-19 Period (n = 262)		*P*
Age (years)	77	(46-99)	71.5	(48-93)	<.001
Male sex	155	(55.4)	135	(51.5)	.372
ECOG PS					
0	3	(1.1)	1	(0.4)	.011
1	166	(59.3)	193	(73.7)	
2	37	(13.2)	25	(9.5)	
3	53	(18.9)	30	(11.5)	
4	21	(7.5)	13	(5.0)	
Stage (TNM)					
1	42	(15.0)	37	(14.1)	.123
2	41	(14.6)	42	(16.1)	
3	48	(17.2)	27	(10.3)	
4	149	(53.2)	156	(59.5)	
Primary tumor location					
Head	145	(51.8)	116	(44.3)	.080
Body or tail	135	(48.2)	146	(55.7)	
Maximum tumor size (mm)	35	(2-120)	35	(3-90)	.061
Symptoms at hospital visit					
Abdominal pain	195	(69.6)	156	(59.5)	.014
Jaundice	73	(26.1)	65	(24.8)	.736
Diabetes mellitus	111	(39.6)	114	(43.5)	.361
Weight loss	62	(22.1)	74	(28.2)	.102
Dyspepsia	88	(31.4)	88	(33.6)	.592
Fatigue	126	(45.0)	99	(37.8)	.089
Laboratory findings					
Hemoglobin (g/dL)	12.5	(6.0-20.4)	12.8	(5.2-17.4)	.028
Albumin (g/dL)	4.0	(2.2-21.1)	4.3	(2.6-28.8)	.009
CA 19-9 (U/mL)	284.7	(0.0-70000.0)	203.7	(0.5-70000.0)	.824
Total bilirubin (mg/dL)	0.8	(0.1-37.0)	0.8	(0.2-35.0)	.883
HbA1C (%)	6.5	(3.0-14.8)	6.6	(0.4-13.3)	.487
C-reactive protein (mg/L)	9.5	(0.0-296.0)	6.8	(0.0-229.0)	.101

Values are presented as numbers (%) or median (range).

COVID-19, coronavirus disease 2019; CA 19-9, carbohydrate antigen 19-9; ECOG, Eastern Cooperative Oncology Group; PS, performance status; TNM, tumor size, lymph node involvement, metastasis.

**Table 2.** Comparison of the Pancreatic Cancer Treatment Modalities Between Pre- and Post-Coronavirus Disease Outbreak Periods

Variables	Pre-COVID-19 Period (n = 280)	COVID-19 Period (n = 262)	*P*
Treatment modality chosen			
Surgery	26 (9.3)	37 (14.1)	<.001
Chemotherapy	67 (23.9)	92 (35.1)	
Best supportive care	141 (50.4)	109 (41.6)	
Referred to another hospital for a second opinion	46 (16.4)	24 (9.2)	
First line chemotherapy*	71 (100.0)	92 (100.0)	<.001
Gemcitabine + nab-paclitaxel	33 (46.5)	38 (41.3)	
FOLFIRINOX^**^	24 (33.8)	53 (57.6)	
Others	14 (19.7)	1 (1.1)	

Values are presented as numbers (%).

COVID-19, coronavirus disease 2019.

*Analyzed only in patients whose treatment choice was chemotherapy.

**The FOLFIRINOX regimen consists of 4 different chemotherapy drugs: fluorouracil, leucovorin, irinotecan, and oxaliplatin.

**Table 3.** Comparison of the Number of Patients with Pancreatic Cancer Stage 3 and 4 of the Pre-Coronavirus Disease 2019 (COVID-19) vs. Post-COVID-19 Periods

Variable	Pre-COVID-19 Period (n = 197)		Post-COVID-19 Period (n = 183)		*χ* ^2^	*P*
	n	%	n	%		
Stage 3	48	24.40	27	14.80	5.53	.019
Stage 4	149	75.60	156	85.20		

Values are presented as numbers (%).

COVID-19, coronavirus disease 2019.

**Table 4.** Comparison of the Characteristics and Treatment Modalities Among Stage 3 and 4 Pancreatic Cancer Cases Between the Pre-Coronavirus Disease (COVID-19) and Post-COVID-19 Periods

Characteristic	Pre-COVID-19 Period (n = 197)		Post-COVID-19 Period (n = 183)		*P*
Age (years)	77	(46-99)	72	(48-93)	<.001
Male sex	113	(57.4)	100	(54.6)	.594
ECOG PS					
0	1	(0.5)	0	(0.0)	.033
1	110	(55.8)	130	(71.0)	
2	27	(13.7)	19	(10.4)	
3	41	(20.8)	22	(12.0)	
4	18	(9.1)	12	(6.6)	
Treatment decision					
Surgery	4	(2.0)	4	(2.2)	.009
Chemotherap y	56	(28.4)	80	(43.7)	
Best supportive care	113	(57.4)	88	(48.1)	
Referred to another hospital for a second opinion	24	(12.2)	11	(6.0)	
Primary tumor location					
Head	85	(43.1)	69	(37.7)	.280
Body or tail	112	(56.9)	114	(62.3)	
Maximum tumor size (mm)	40	(10-120)	39	(10-90)	.067
Symptoms at a hospital visit					
Abdominal pain	145	(73.6)	119	(65.0)	.070
Jaundice	42	(21.3)	38	(20.8)	.895
Diabetes mellitus	79	(40.1)	76	(41.5)	.777
Weight loss	50	(25.4)	59	(32.2)	.140
Dyspepsia	65	(33.0)	64	(35.0)	.684
Fatigue	96	(48.7)	70	(38.3)	.040
Laboratory findings					
Hemoglobin (g/dL)	12.4	(6.0-20.4)	12.8	(5.2-16.9)	.017
Albumin (g/dL)	4.0	(2.2-5.0)	4.3	(2.6-4.9)	<.001
CA 19-9 (U/mL)	593.3	(0.0-70000.0)	431.5	(0.5-70000.0)	.882
Total bilirubin (mg/dL)	0.8	(0.1-37.0)	0.8	(0.2-34.5)	.827
HbA1C (%)	6.5	(3.0-14.8)	6.5	(0.4-13.3)	.997
C-reactive protein (mg/L)	14.5	(0.0-296.0)	6.3	(0.0-229.0)	.036
First line chemotherapy^*^					
Gemcitabine + nab-paclitaxel	31	(52.5)	36	(45.0)	<.001
FOLFIRINOX^**^	17	(28.8)	44	(55.0)	
Others	11	(18.6)	0	(0.0)	

Values are presented as numbers (%) or median (range).

CA 19-9, carbohydrate antigen 19-9; COVID-19, coronavirus disease 2019; ECOG, Eastern Cooperative Oncology Group; PS, performance status; TNM, tumor size, lymph node involvement, metastasis.

*Analyzed only in patients whose treatment choice was chemotherapy.

**The FOLFIRINOX regimen consists of 4 different chemotherapy drugs: fluorouracil, leucovorin, irinotecan, and oxaliplatin.

**Table 5.** Univariate and Multivariate Analysis of Prognostic Factors for Survival of Patients with Pancreatic Cancer

Prognostic Factor	Univariate Analysis			Multivariate Analysis		
	Hazard Ratio	95% CI	*P*	Hazard Ratio	95% CI	*P*
Age, years						
≤59 years	Reference					
60-69	0.912	0.648-1.283	.597			
70-79	1.325	0.956-1.839	.091			
≥80	2.243	1.632-3.083	<.001	1.049	0.659-1.669	.841
ECOG PS						
0-1	Reference					
2-4	2.635	2.169-3.201	<.001	2.103	1.460-3.030	<.001
Sex						
Male	Reference					
Female	1.097	0.910-1.323	.333			
Diagnosis period						
Pre-COVID	Reference					
COVID	0.867	0.717-1.047	.139			
Tumor location						
Head	Reference					
Body/tail	1.164	0.966-1.403	.111			
Stage						
I	Reference					
II	2.933	1.949-4.414	<.001			
III	2.695	1.761-4.124	<.001			
IV	5.187	3.616-7.441	<.001	1.895	1.169-3.074	.010
CA 19-9						
Elevated^#^	1.407	1.129-1.754	.002	0.987	0.724-1.345	.932
Hemoglobin						
Decreased^#^	1.683	1.387-2.041	<.001	1.663	1.239-2.232	.001
Albumin						
Decreased^#^	1.806	1.382-2.360	<.001	0.592	0.365-0.961	.034
Total bilirubin						
Elevated^#^	1.057	0.871-1.283	.575			
HbA1C						
Elevated^#^	1.065	0.824-1.376	.629			
C-reactive protein						
Elevated^#^	1.669	1.377-2.022	<.001	1.392	1.059-1.828	.018
Treatment						
Surgery	Reference					
Chemotherapy	3.423	2.243-5.225	<.001	2.063	1.180-3.608	.011
Best supportive care	7.151	4.743-10.784	<.001	2.791	1.556-5.005	.001
Referred to another hospital	2.379	1.475-3.811	<.001	1.374	0.739-2.555	.315
Abdominal pain	1.301	1.068-1.584	.009	1.224	0.923-1.624	.161
Jaundice	1.065	0.861-1.317	.561			
Diabetes mellitus	0.897	0.742-1.084	.260			
Weight loss	0.927	0.746-1.153	.497			
Dyspepsia	1.078	0.884-1.315	.458			
Fatigue	1.711	1.417-2.065	<.001	1.019	0.736-1.409	.912

CA 19-9, carbohydrate antigen 19-9; COVID-19, coronavirus disease 2019; ECOG, Eastern Cooperative Oncology Group; PS, performance status.

^#^The reference ranges are as follows: CA 19.9 ≤ 34 U/mL, hemoglobin ≥ 13.0 g/dL, albumin ≥ 3.5 g/dL, total bilirubin ≤ 1.2 mg/dL, HbA1C ≤ 5.9%, and C-reactive protein ≤ 5.0 mg/L.
